# Ultra Thin Poly-Si Nanosheet Junctionless Field-Effect Transistor with Nickel Silicide Contact

**DOI:** 10.3390/ma10111276

**Published:** 2017-11-07

**Authors:** Yu-Ru Lin, Wan-Ting Tsai, Yung-Chun Wu, Yu-Hsien Lin

**Affiliations:** 1 Department of Engineering and System Science, National Tsing Hua University, Hsinchu 30013, Taiwan; flywing79222@gmail.com (Y.-R.L.); ycwu@ess.nthu.edu.tw (Y.-C.W.); 2 Department of Electrical Engineering, National United University, Miao-Li 36063, Taiwan; tinayork123@gmail.com

**Keywords:** nickel silicide, junctionless field-effect transistor, ultra thin body

## Abstract

This study demonstrated an ultra thin poly-Si junctionless nanosheet field-effect transistor (JL NS-FET) with nickel silicide contact. For the nickel silicide film, two-step annealing and a Ti capping layer were adopted to form an ultra thin uniform nickel silicide film with low sheet resistance (Rs). The JL NS-FET with nickel silicide contact exhibited favorable electrical properties, including a high driving current (>10^7^A), subthreshold slope (186 mV/dec.), and low parasitic resistance. In addition, this study compared the electrical characteristics of JL NS-FETs with and without nickel silicide contact.

## 1. Introduction

Metal silicide techniques have been developed at the deep submicron level in microelectronics as contact materials to the source, drain, and gate regions [1,2,3]. Nickel silicides are valuable electronic materials used as contacts for field-effect transistors, as interconnects, and in nanoelectronic devices because of their source/drain (S/D) sheet resistance. To replace the currently used cobalt silicide and titanium silicide, nickel silicide has attracted attention as a candidate for future nodes because of advantages including low temperature processing, low silicon consumption, no bridging failure property, low mechanical stress, and low contact resistance [4,5,6]. Notably, the diffusion-controlled reaction of Ni silicidation formation occurs by Ni diffusion into the silicon area. The Ni vacancies generated by themselves are mainly located in the Ni layer instead of in the Si layer [3,7]. As device dimensions are scaled down, lowering sheet resistance is a critical factor in applications where silicides are used. Nickel silicide has some limitations, such as (1) interfacial silicon oxide present at the Ni/Si interface hampering the silicidation between Ni and Si, (2) the thermal stability of nickel silicide, and (3) the need to reduce the junction leakage current of nickel-silicided junctions [8,9].

A Ti capping layer can prevent oxygen adsorption on deposited nickel silicide [10,11,12], and can also prevent oxidation during the silicidation process because it is an excellent oxygen scavenger. Other advantages of Ti capping include increased uniformity and thermal stability, as well as a low junction leakage current. Furthermore, the development of metal silicide technology through two-step rapid thermal annealing (RTA) has been extensively investigated as a low resistance contact [13,14]. The first step involves low temperature annealing to drive nickel to diffuse into Si, to thus form a nickel-rich silicide layer. The second step involves higher temperature annealing to transform the nickel silicide phase.

A junctionless field-effect transistor (JL-FET) can be fabricated simply by heavily doping the channel and S/D regions simultaneously. Because of the special doping profile, JL-FETs have several advantages such as a low thermal budget that can integrate with high-k/metal-gate more easily than can conventional metal–oxide–semiconductor field-effect transistors (MOSFETs), a longer effective channel length than conventional MOSFETs, and the avoidance of complicated S/D engineering. To solve the JL-FET turn-off problem, an ultrathin body structure is required to achieve a fully-depleted channel region in the off state [15,16,17]. However, the drive current (I_D_) declines as transistor features are scaled. Therefore, the study presents an ultra thin poly-Si junctionless nanosheet field-effect transistor (JL NS-FET) with a nickel silicide contact. Moreover, this study presents results obtained from nickel silicide that indicate the state of the substrate and the silicide phase. Nickel silicide quality was analyzed using X-ray diffraction (XRD), transmission-electron microscopy (TEM), and a four-point probe (FPP).

## 2. Silicide Film Analysis

**Figure 1:**
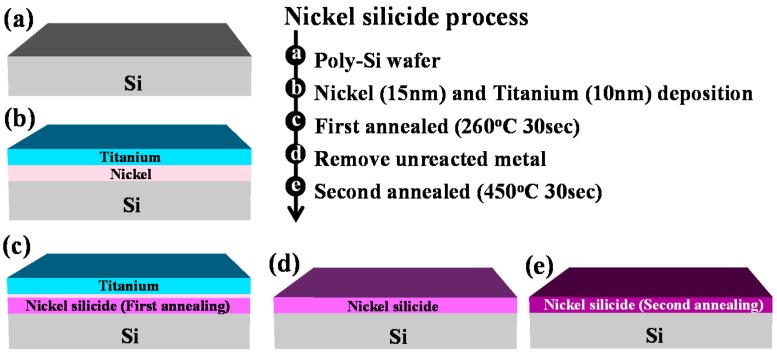
Key process flow diagram and schematics of nickel silicide film.

**Figure 2:**
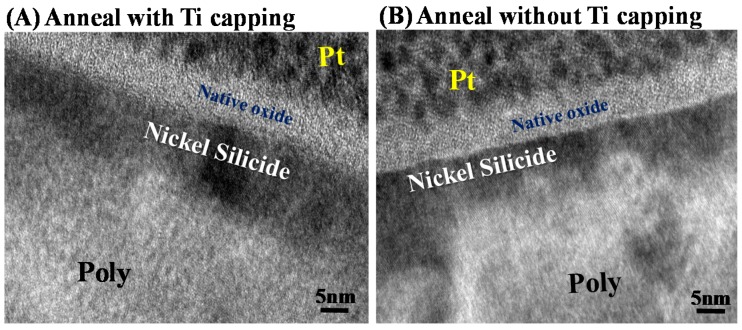
TEM images for annealing process (**A**) with and (**B**) without titanium capping on nickel.

Figure 1 displays the key process flow diagram and schematics of nickel silicide film fabrication. The nickel silicide film was fabricated by initially depositing a 50-nm-thick layer of undoped amorphous Si (a-Si) through low pressure chemical vapor deposition (LPCVD) at 550 °C. A poly-Si layer was then formed through solid-phase recrystallization (SPC) at 600 °C for 24 h [18]. Subsequently, a 15-nm-thick nickel film and 10-nm-thick titanium film were deposited through physical chemical deposition (PVD). The films were first annealed through rapid thermal annealing at 260 °C for 30 s to form a nickel silicide film. The unreacted nickel and titanium films were removed by selective etching with sulfuricacid solution at 120 °C. In the second step, rapid thermal annealing was performed at 450 °C for 30 s to lower the sheet resistance and firmly merge the phase (Figure 4). Figure 2 displays high-resolution TEM images for the first annealing process with and without titanium capping. As shown in Figure 2A, the nickel silicide film formed through the first annealing with Ti capping was uniform because the Ti capping layer suppressed the oxidation of the nickel silicide films. This serves as evidence that a Ti capping layer is an oxygen scavenger. Oxygen is a pollutant in the silicide process and reduces the quality of nickel silicide film, as indicated in Figure 2B.

**Figure 3:**
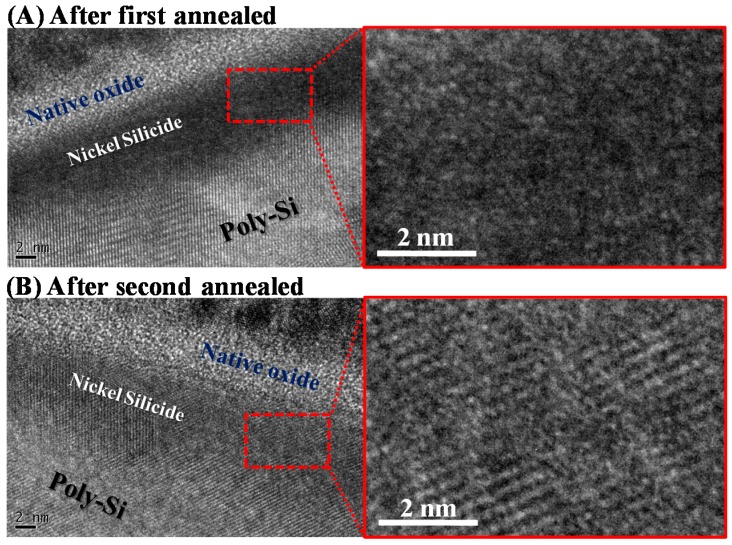
TEM images for nickel silicide film after (**A**) the first and (**B**) the second annealing processes.

**Figure 4:**
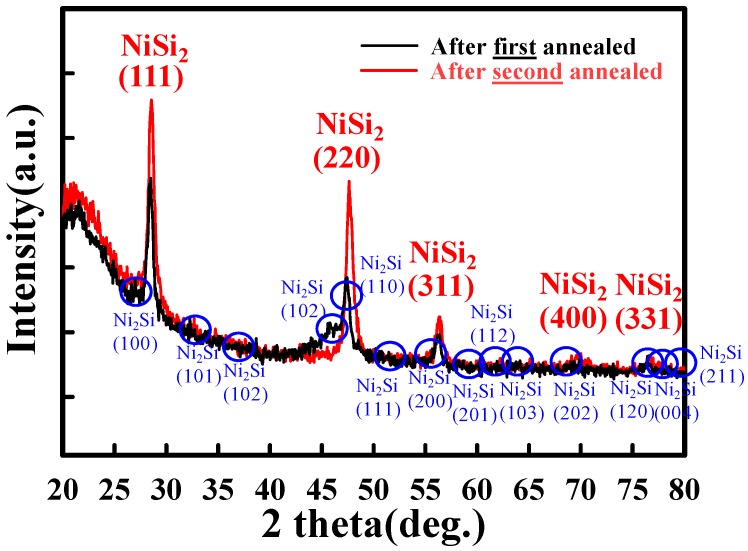
XRD phase analysis for the nickel silicide film after the first and second annealing processes.

Figure 3 displays TEM images of the nickel silicide film after the first and second annealing processes. The second annealing, which exhibited a superior crystal phase than the first annealing, was utilized to lower the sheet resistance and steadily merge the phase (Figure 4). The FPP revealed that the sheet resistance of nickel silicide film was 462.8 ± 20 Ω/sq after the first annealing and 108.7 ± 1 Ω/sq after the second annealing. Figure 4 shows the phase of the nickel silicide film captured by XRD, revealing that the degradation at the second annealing was a result of the phase transformation of Ni_2_Si to NiSi_2_ and agglomeration. Thus, after the second annealing, nickel was redistributed in the silicide instead of the underlying silicon being penetrated.

## 3. Device Structure and Fabrication

**Figure 5:**
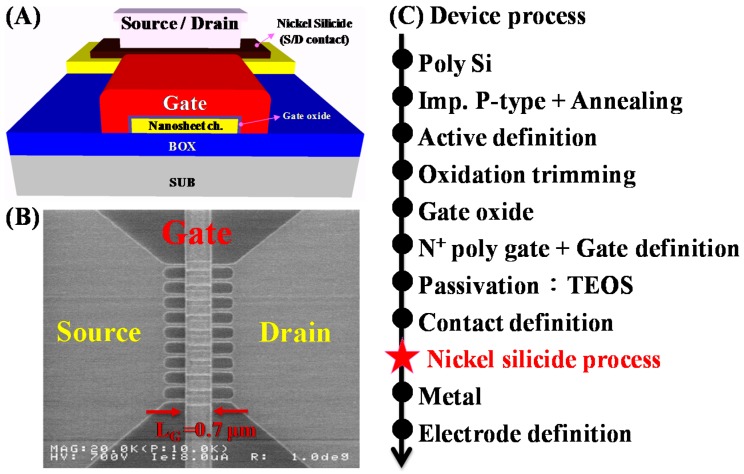
(**A**) Schematics of a single nanosheet cross-section; (**B**) Top-view SEM image of the junctionless nanosheet field-effect transistor (JL NS-FET) structure with 10 nanosheets; (**C**) Process flow of the JL NS-FET.

Figure 5A presents a schematic of a single nanosheet cross-section of an ultra thin poly-Si JL NS-FET with a nickel silicide contact. A conventional JL NS-FET without nickel silicide contact was also fabricated for comparison. Figure 5B shows a top-view scanning electron microscope (SEM) image of the 10-strip nanosheets active region with a gate length of 0.7 µm. The gate covers 10 nanosheet strips with a printed width of 100 nm, which formed a sheet-shaped structure. Figure 5C displays the process flow for fabricating the JL NS-FET. The device was fabricated by initially growing a 400-nm-thick thermal SiO_2_ layer on a 6-inch Si wafer. In addition, a 50-nm-thick layer of undoped a-Si was deposited through LPCVD at 550 °C. The poly-Si layer was then formed using the SPC method at 600 °C for 24 h. The p-channel of the poly-Si layer was implanted with BF_2_ ions at a dose of 2 × 10^14^ cm^−2^ by 30 keV. The dopant was then activated through furnace annealing at 600 °C for 4 h in nitrogen ambient. The nanosheet channels were patterned using e-beam lithography (EBL) and transferred using reactive-ion etching (RIE).

To form the ultra thin poly-Si nanosheet channel, the naked nanosheets were first thermally oxidized and the oxide layer was subsequently removed through hydrogen fluoride dipping. After active region patterning, an 8-nm-thick thermal oxide layer was deposited as the gate oxide layer. A 150-nm-thick in-situ n^+^ poly-Si deposition was performed as a gate electrode and patterned using EBL and RIE. Subsequently, a 250-nm-thick passivation layer of tetraethoxysilane (TEOS) was deposited. After contact region patterning through EBL and RIE, a 15-nm nickel film and 10-nm titanium film were deposited using PVD. The device was then annealed through rapid thermal annealing (RTA) at 260 °C for 30 s and the unreacted nickel film and titanium film were selectively etched using wet etching for 10 min at 120 °C. RTA was performed a second time at 450 °C for 30 s to lower the sheet resistance and firmly merge the phase. Finally, 300-nm-thick Al–Si–Cu metallization was performed and sintered at 400 °C for 30 min.

## 4. Results and Discussion

**Figure 6:**
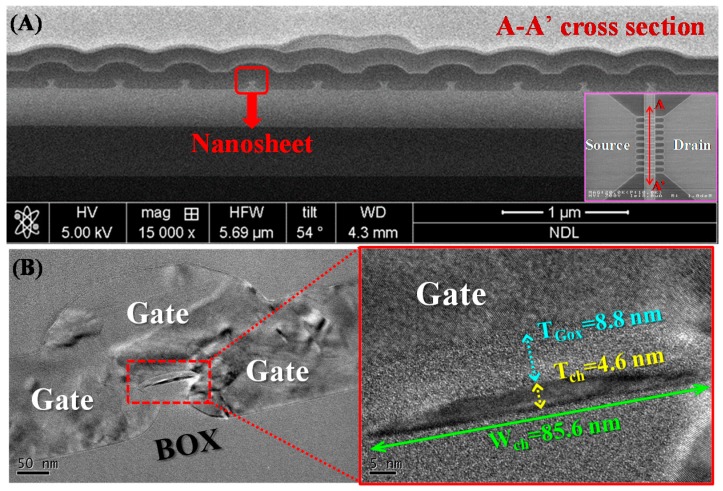
(**A**) Focused ion beam (FIB) section of the SEM image of a cross-sectional view of the JL NS-FET; (**B**)TEM image of the single nanosheet cross-sectional structure and magnified image.

Figure 6A displays across-sectional focused ion beam (FIB) image of the JL NS-FET along the gate direction (A–A’), revealing the 10-strip nanosheet structure. Figure 6B displays TEM images of the nanosheet channel perpendicular to the gate direction of the JL NS-FET. The poly-Si nanosheet assumed the form of a sheet and was surrounded by the N^+^ poly-Si gate to form a tri-gate structure. The magnified image of the single nanosheet reveals that the width and height were 85.6 and 4.6 nm, respectively. The effective width (W_eff_) for the JL NS-FET was 85.6 × 10 nm. The gate oxide thickness of the JL NS-FET was approximately 8.8 nm.

**Figure 7:**
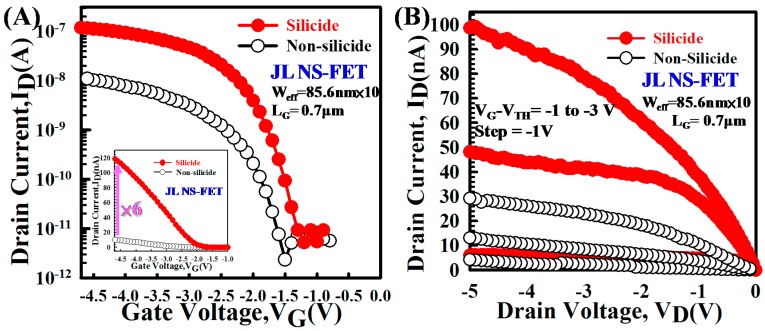
(**A**) I_D_–V_G_ curves of the silicide and nonsilicide JL NS-FETs. The linear scale of the I_D_–V_G_ curves indicates that the silicide JL NS-FET had 6 times the saturation current of the nonsilicide device(the inset displays the linear scale); (**B**) Transfer I_D_–V_D_ curves of the silicide and nonsilicide JL NS-FETs for various V_G_−V_TH_ values.

**Figure 8:**
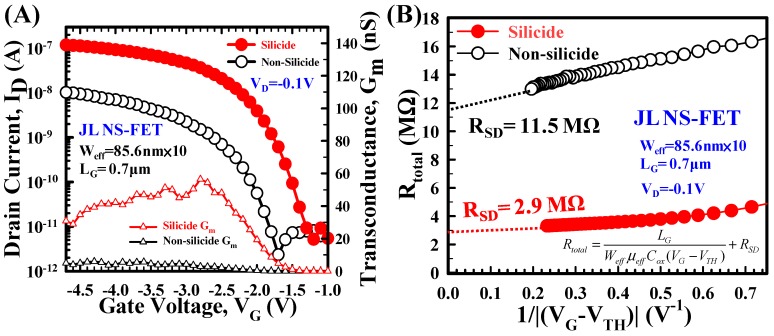
(**A**) I_D_–V_G_ curves and G_m_ of the silicide and nonsilicide JL NS-FETs. The G_m_ value of the silicide device is larger than that of the nonsilicide device; (**B**)Total resistance (R_total_) of the silicide and nonsilicide devices as a function of gate voltage at V_D_ = 0.1 V.

Figure 7A exhibits the I_D_–V_G_ curves of the JL NS-FET with a nickel silicide contact (silicide) and without (nonsilicide). The subthreshold slopes of the silicide and nonsilicide JL NS-FET were 186 mV/dec. and 194 mV/dec., and the corresponding threshold voltages (V_TH_) were −1.8 V and −2.3 V, where V_TH_ refers to the gate voltage at I_D_ = 10^−^^9^. The silicide JL NS-FET had a superior I_on_/I_off_ current ratio (2.9 × 10^4^) than did the nonsilicide device. The linear scale of the I_D_–V_G_ curves shows that the silicide JL NS-FET had six times the saturation current of its nonsilicide counterpart (the inset in Figure 7A displays the linear scale). Figure 7B plots the I_D_–V_D_ characteristics of the silicide and nonsilicide JL NS-FETs. The saturation current of the silicide device was approximately 3.25 times that of the nonsilicide device at V_G_ − V_TH_ = −3 V and V_D_ = −3 V. The silicide device had a higher saturation current because of its lower S/D parasitic resistance, as shown in Figure 8B.

Figure 8A demonstrates the transfer I_D_–V_G_ characteristics and transconductance (G_m_) of the silicide and nonsilicide JL NS-FETs at V_D_ = −0.1 V. The maximum G_m_ values were 56.5 and 9 nS at V_G_ = −2.8 V for the silicide and nonsilicide devices, respectively. The field-effect mobilities of the silicide and nonsilicide devices calculated from the maximum G_m_ at V_D_ = −0.1 V were 8.4 and 1.3 cm^2^/Vs, respectively. Figure 8B displays the total resistance (R_total_) curves calculated based on the I_D_–V_G_ characteristics. The R_SD_ of the silicide and nonsilicide contact devices were 2.9 and 11.5 MΩ where the *X*-axis was equal to 0, respectively.

## 5. Conclusions

An ultra thin poly-Si JL NS-FET with nickel silicide contact was demonstrated successfully, exhibiting a high driving current (>10^7^ Å), subthreshold slope (186 mV/dec.), and low parasitic resistance. In the silicide film analysis, the second annealing step was applied to lower the sheet resistance and firmly merged the phase of the silicide film. Additionally, we used a Ti capping layer to remove interfacial oxides on the Si surface and promote a silicidation reaction between Ni and Si. In summary, the JL NS-FET with nickel silicide contact exhibited competitive short-channel behavior and improved drive current.
